# A nomogram for prediction of deep venous thrombosis risk in elderly femoral intertrochanteric fracture patients: A dual-center retrospective study

**DOI:** 10.3389/fsurg.2022.1028859

**Published:** 2023-01-06

**Authors:** Guangheng Xiang, Xiaoyu Dong, Shenglei Lin, Leyi Cai, Feiya Zhou, Peng Luo, Juanjuan Zhu

**Affiliations:** ^1^ Department of Orthopaedic, The Second Affiliated Hospital and Yuying Children's Hospital of Wenzhou Medical University, Wenzhou, China; ^2^ School of Pharmaceutical Science, Wenzhou Medical University, Wenzhou, China; ^3^ Department of Orthopaedic, Wenzhou Central Hospital, Wenzhou, China; ^4^ Department of Geriatrics and Neurology, The Second Affiliated Hospital and Yuying Children's Hospital of Wenzhou Medical University, Wenzhou, China

**Keywords:** nomogram, deep venous thrombosis, risk factors, elderly, femoral intertrochanteric fracture

## Abstract

**Objective:**

Deep venous thrombosis (DVT) of the lower extremity is a common perioperative complication of femoral intertrochanteric fracture. This study aimed to identify the risk factors of lower extremity deep vein thrombosis (DVT) in elderly femoral intertrochanteric fracture patients and establish a nomogram model.

**Methods:**

From August 2014 to June 2021, a total of 1,652 femoral intertrochanteric fracture patients over the age of 65 were enrolled in our study. We distinguished independent risk factors by univariate and multivariate Cox analyses. A nomogram model was then built, and the discriminative and calibration of the model was evaluated through receiver operating characteristics (ROC) and calibration plots.

**Results:**

A total of 378 patients developed DVT (292 in the training group, 86 in the validation group) while the remaining patients did not. According to the univariate and multivariate Cox analyses results, age (OR = 1.07, 95% CI: 1.04–1.10), fibrinogen (OR = 2.09, 95% CI: 1.68–2.60), D-dimer (OR = 1.33, 95% CI: 1.27–1.40), time from injury to admission (OR = 1.78, 95% CI: 1.55–2.05), functional status (OR = 4.21, 95% CI: 2.86–6.20), and diabetes (OR = 1.65, 95% CI: 1.10–2.48) were identified as independent risk factors of DVT. The ROC values for DVT of the training and validation group were 0.862 and 0.912, and the *P*-value of the Hosmer-Lemeshow calibration test was 0.767.

**Conclusion:**

This nomogram model can be used to predict the probability of preoperative DVT in elderly patients with femoral intertrochanteric fracture and guide physician in perioperative thrombosis management.

## Introduction

Osteoporotic hip fractures in the elderly have become a major public health problem. With the increase in the elderly population, the incidence is on the rise (1). The annual incidence of hip fractures worldwide is estimated to increase from 1.6 million in 2,000 to at least 4.5 million in 2,050 (2). Elderly patients with femoral intertrochanteric fractures have high mortality, many complications, and decreased ability to take care of themselves in daily life, which has become a serious disease that needs to be solved urgently (3–5).

Deep venous thrombosis (DVT) of the lower extremity is a common perioperative complication of femoral intertrochanteric fracture (6–8). DVT of the lower limb mainly manifests as pain, swelling, and increased soft tissue tension in the affected limb, and there are often tenderness, Homans sign, and Neuhof sign at the thrombus site. After the DVT of the lower extremity falls off, it can flow into the pulmonary artery and cause pulmonary embolism (PE), endangering the life of the patient (9,10). Therefore, early prevention, diagnosis and treatment are of great significance.

Venography is one of the methods to diagnose deep vein thrombosis, but it is invasive and non-repeatable (11). Ultrasonography is becoming more and more popular in clinical practice, and many guidelines regard it as the gold standard for diagnosis of DVT, however it requires patients to change their position, aggravating pain and injury (12). In addition, the diagnostic value of plasma markers such as D-dimer in DVT patients is controversial (13–16). D-dimer was tested for outpatients and not inmates and does not work for high risk patients.

This study explored the risk factors of perioperative lower extremity DVT in elderly patients with femoral intertrochanteric fracture, and established a nomogram model to predict the incidence. Provide reference for the early prevention and diagnosis of high-risk DVT patients in the future.

## Materials and methods

### Inclusion and exclusion criteria

This study followed the guidelines of the “Declaration of Helsinki” and was approved by the ethics committee of our hospital (The Second Affiliated Hospital and Yuying Children's Hospital of Wenzhou Medical University, Wenzhou, China, NO. L-2020–22). All data is analyzed anonymously, and personal identifiers are completely deleted.

We retrospectively collected data on elderly femoral intertrochanteric fracture patients in our hospital and Wenzhou Central Hospital from August 2014 to June 2021. The eligibility criteria are: 1) unilateral femoral intertrochanteric fractures (International Classification of Diseases, 10th Edition [ICD-10] S72.1); 2) low-energy injuries (for example, falling from standing height and osteoporosis); 3) age ≥65 years; 4) recipients undergoing open or closed reduction and internal fixation; 5) complete clinical data. Exclusion criteria include: 1) pathological fracture; 2) multiple fractures or multiple traumas; 3) previous hip fracture; 4) open fracture; 5) conservative treatment; 6) patients with poor compliance such as mental illness; 7) contraindications to the use of anticoagulant drugs; 8) in the acute phase of cardiovascular and cerebrovascular diseases. Finally, patients in our hospital were enrolled in the training group and patients from Wenzhou Central Hospital were enrolled in the validation group.

### Diagnosis and treatment of DVT

We used Doppler ultrasonography to diagnose DVT. Color Doppler ultrasonography was performed by experienced radiologists and diagnoses according to the criteria (17). All patients underwent vascular ultrasonography examination of both lower extremities before surgery.

For all patients without contraindications to anticoagulation, LMWH was subcutaneously injected to prevent DVT. For patients with peripheral type DVT, a therapeutic anticoagulation protocol was implemented based on the consultation advice of vascular surgeon. However, for patients who were contraindicated to use anticoagulant drugs or central or mixed DVT, inferior vena cava filter was used to prevent fatal pulmonary embolism.

### Analysis of risk factors

In our study, patients were characterized at baseline in terms of age, gender, body mass index (BMI), hemoglobin, blood platelet, serum albumin, c-reactive protein (CRP), fibrinogen, D-dimer, prothrombin time (PT), activated partial thromboplastin time (APTT), functional status, current smoking status, current drinking status and time from injury to admission. The patient's previous medical history was evaluated, including hypertension, diabetes, coronary heart disease, heart failure, cerebrovascular disease, thrombosis and malignancy. Factors related to surgery was also assessed, including operative time, blood transfusion, anesthesia type, American Society of Anesthesiologists (ASA) classification.

### Statistical analysis

The Kolmogorov-Smirnov method was used to test the distribution status of measurement data. The measurement data conforming to the normal distribution were expressed as the means ± standard deviations, and the independent sample *t*-test was used for the comparison between groups. The enumeration data was expressed as *n* (%), and the comparison between groups was performed by the chi-square test. Univariate analysis was used to assess the association between different variables and DVT. Then, variables with significance *P* < 0.1 were subjected to multivariate analysis to determine the independent risk factors. Finally, we established a nomogram prediction model for DVT based on the regression coefficients of the independent risk factors.

The discrimination of dichotomous results was usually evaluated by calculating the area under the curve (AUC) of the receiver operating characteristic (ROC) curve. Generally, a prediction model with an AUC of 0.5–0.75 is considered acceptable, and AUC > 0.75 means that the model shows excellent discriminative power. The calibration curve was the image comparison of predicting probabilities and actual probabilities. The closer the predicted probabilities to the standard curve, the better the conformity of the model is. In addition, the decision curve analysis was carried out to assess the net benefit of the nomogram to the decision. Statistical analyses were carried out using R version 3.6.1 for Windows (R Foundation for Statistical Computing, Vienna, Austria) and Empower Stats (http://www.empowerstats.com, X & Y Solutions, Inc., Boston, MA). *P* value less than 0.05 was considered statistically significant.

## Results

From August 2014 to June 2021, 1,652 patients with femoral intertrochanteric fractures over 65 years of age who underwent surgical treatment were the subjects of this study. Among them, 1,268 patients from our hospital were included in the training group, and 384 patients from Wenzhou Central Hospital were included in the validation group. The baseline data of patients from the training group and the validation group were analyzed, and no significant difference was found between the two groups (*P* > 0.05).

In the training group, 292 (23.03%) patients developed DVT with an average age of 84.11 ± 5.91 years, and 976 (76.97%) patients did not develop DVT with an average age of 78.82 ± 7.25 years (*P* < 0.001). Similar results appeared in the validation group, 86 (22.40%) patients developed DVT with an average age of 80.86 ± 7.91 years, and 298 (77.60%) patients did not develop DVT with an average age of 78.28 ± 7.97 years (*P* = 0.008). In the training group, the fibrinogen and D-dimer of DVT patients was 4.04 ± 0.84 and 9.50 ± 4.14, whereas the fibrinogen and D-dimer of non-DVT patients was 3.48 ± 0.73 and 6.47 ± 2.87 (*P* < 0.001). In the validation group, the fibrinogen of DVT and non-DVT patients was 4.20 ± 0.80 and 3.18 ± 0.72, the D-dimer of DVT and non-DVT patients was 9.17 ± 3.77 and 5.90 ± 3.15 (*P* < 0.001). In the training and validation group, the time from injury to admission of DVT patients was longer than that of non-DVT patients (2.28 ± 1.34 and 2.29 ± 1.28, respectively). As shown in Table 1, we found that the ASA classification, functional status and diabetes history were statistically different between DVT and non-DVT (*P* < 0.05).

**Table 1:** Baseline characteristics.

Variable	Development group (*n* = 1268)			Validation group (*n* = 384)		
	Without DVT (*n* = 976)	With DVT (*n* = 292)	*P*-value*	Without DVT (*n* = 298)	With DVT (*n* = 86)	*P*-value*
Age, years	78.82 ± 7.25	84.11 ± 5.91	<0.001	78.28 ± 7.97	80.86 ± 7.91	0.008
BMI, Kg/m^2^	22.17 ± 2.61	21.99 ± 2.91	0.057	22.57 ± 2.94	22.71 ± 2.96	0.716
Hemoglobin, g/L	105.62 ± 13.59	106.24 ± 13.10	0.356	109.88 ± 13.49	108.76 ± 13.11	0.570
Serum albumin, g/dL	36.48 ± 3.67	36.51 ± 3.79	0.399	37.07 ± 4.21	37.43 ± 4.64	0.561
C-reactive protein, mg/L	53.49 ± 24.26	53.70 ± 23.69	0.389	51.09 ± 23.70	52.09 ± 24.18	0.670
Blood platelet,10^9^/L	190.62 ± 55.99	183.49 ± 50.02	0.245	187.13 ± 53.34	181.62 ± 51.27	0.439
Fibrinogen, g/L	3.48 ± 0.73	4.04 ± 0.84	<0.001	3.18 ± 0.72	4.20 ± 0.80	<0.001
D-dimer, μg/ml	6.47 ± 2.87	9.50 ± 4.14	<0.001	5.90 ± 3.15	9.17 ± 3.77	<0.001
PT, s	13.07 ± 0.81	13.04 ± 1.33	0.078	12.82 ± 0.83	12.83 ± 1.17	0.421
APTT, s	34.29 ± 4.44	34.86 ± 4.87	0.130	34.72 ± 4.19	33.89 ± 4.59	0.143
Operative time, min	51.94 ± 10.73	51.14 ± 11.83	0.186	52.20 ± 9.96	51.08 ± 9.03	0.395
Time from injury to admission, days	0.55 ± 0.91	1.28 ± 1.34	<0.001	0.50 ± 0.91	1.29 ± 1.28	<0.001
Gender			0.619			0.795
Male	343 (35.14%)	98 (33.56%)		112 (37.58%)	31 (36.05%)	
Female	633 (64.86%)	194 (66.44%)		186 (62.42%)	55 (63.95%)	
Anesthesia type			0.082			0.271
Regional	663 (67.93%)	214 (73.29%)		207 (69.46%)	65 (75.58%)	
General	313 (32.07%)	78 (26.71%)		91 (30.54%)	21 (24.42%)	
ASA classification			0.002			0.021
1	103 (10.55%)	25 (8.56%)		24 (8.05%)	8 (9.30%)	
2	460 (47.13%)	114 (39.04%)		148 (49.66%)	36 (41.86%)	
3	340 (34.84%)	113 (38.70%)		106 (35.57%)	27 (31.40%)	
4	73 (7.48%)	40 (13.70%)		20 (6.71%)	15 (17.44%)	
Functional status			<0.001			<0.001
Independent	857 (87.81%)	193 (66.10%)		275 (92.28%)	57 (66.28%)	
Dependent	119 (12.19%)	99 (33.90%)		23 (7.72%)	29 (33.72%)	
Smoking			0.224			0.245
No	624 (63.93%)	198 (67.81%)		184 (61.74%)	59 (68.60%)	
Yes	352 (36.07%)	94 (32.19%)		114 (38.26%)	27 (31.40%)	
Drinking			0.168			0.353
No	748 (76.64%)	235 (80.48%)		221 (74.16%)	68 (79.07%)	
Yes	228 (23.36%)	57 (19.52%)		77 (25.84%)	18 (20.93%)	
Blood transfusion			0.483			0.825
No	709 (72.64%)	206 (70.55%)		215 (72.15%)	61 (70.93%)	
Yes	267 (27.36%)	86 (29.45%)		83 (27.85%)	25 (29.07%)	
Diabetes			0.019			0.030
No	823 (84.32%)	229 (78.42%)		263 (88.26%)	68 (79.07%)	
Yes	153 (15.68%)	63 (21.58%)		35 (11.74%)	18 (20.93%)	
Hypertension			0.266			0.104
No	711 (72.85%)	203 (69.52%)		230 (77.18%)	59 (68.60%)	
Yes	265 (27.15%)	89 (30.48%)		68 (22.82%)	27 (31.40%)	
Coronary heart disease			0.423			0.139
No	900 (92.21%)	265 (90.75%)		288 (96.64%)	80 (93.02%)	
Yes	76 (7.79%)	27 (9.25%)		10 (3.36%)	6 (6.98%)	
Heart failure			0.238			0.478
No	915 (93.75%)	268 (91.78%)		286 (95.97%)	81 (94.19%)	
Yes	61 (6.25%)	24 (8.22%)		12 (4.03%)	5 (5.81%)	
Cerebrovascular disease			0.097			0.298
No	905 (92.73%)	262 (89.73%)		277 (92.95%)	77 (89.53%)	
Yes	71 (7.27%)	30 (10.27%)		21 (7.05%)	9 (10.47%)	
Thrombosis			0.119			0.115
No	886 (90.78%)	256 (87.67%)		276 (92.62%)	75 (87.21%)	
Yes	90 (9.22%)	36 (12.33%)		22 (7.38%)	11 (12.79%)	
Malignancy			0.110			0.909
No	940 (96.31%)	275 (94.18%)		285 (95.64%)	82 (95.35%)	
Yes	36 (3.69%)	17 (5.82%)		13 (4.36%)	4 (4.65%)	

Data are presented as the mean and the standard deviation with the range in parenthesis or expressed as the number with the percentage in parenthesis.

* *P*-value, differences between patients with pneumonia and control. ASA score, american society of anesthesiologists score; BMI, body mass index; PT, prothrombin time; APTT, activated partial thromboplastin time.

A univariate analysis of the training group showed that the significant risk factors were age, fibrinogen, D-dimer, time from injury to admission, ASA classification, functional status and diabetes (*P* < 0.05). The statistically significant variables selected from the univariate analysis were included in the multivariate logistic regression analysis. Ultimately, age (OR = 1.07, 95% CI: 1.04–1.10), fibrinogen (OR = 2.09, 95% CI: 1.68–2.60), D-dimer (OR = 1.33, 95% CI: 1.27–1.40), time from injury to admission (OR = 1.78, 95% CI: 1.55–2.05), functional status (OR = 4.21, 95% CI: 2.86–6.20), and diabetes (OR = 1.65, 95% CI: 1.10–2.48) were identified as independent risk factors for DVT in elderly hip fracture patients (Table 2).

**Table 2:** Univariate and multivariate logistic regression of predictors for DVT.

Variable	Univariate analysis		Multivariate analysis	
	OR (95% CI)	*P* value	OR (95% CI)	*P* value
Age, years	1.12 (1.09, 1.14)	<0.001	1.07 (1.04, 1.10)	<0.001
Gender	1.07 (0.81, 1.41)	0.619		
BMI, Kg/m^2^	0.98 (0.93, 1.02)	0.318		
Hemoglobin, g/L	1.00 (0.99, 1.01)	0.490		
Serum albumin, g/dL	1.00 (0.97, 1.04)	0.909		
C-reactive protein, mg/L	1.00 (0.99, 1.01)	0.899		
Blood platelet,10^9^/L	1.00 (1.00, 1.00)	0.510		
Fibrinogen, g/L	2.47 (2.07, 2.94)	<0.001	2.03 (1.63, 2.54)	<0.001
D-dimer, μg/ml	1.29 (1.24, 1.34)	<0.001	1.34 (1.27, 1.41)	<0.001
PT, s	0.98 (0.85, 1.12)	0.736		
APTT, s	1.03 (1.00, 1.06)	0.059	0.90 (0.77, 1.06)	0.217
Operative time, min	0.99 (0.98, 1.01)	0.277		
Anesthesia type	0.77 (0.58, 1.03)	0.083	0.8 (0.55, 1.15)	0.226
ASA classification	2.26 (1.26, 4.04)	0.006	0.74 (0.34, 1.59)	0.435
Blood transfusion	1.11 (0.83, 1.48)	0.484		
Functional status	3.69 (2.71, 5.03)	<0.001	4.28 (2.90, 6.32)	<0.001
Smoking	0.84 (0.64, 1.11)	0.224		
Drinking	0.80 (0.57, 1.10)	0.168		
Time from injury to admission, days	1.76 (1.56, 1.98)	<0.001	1.79 (1.56, 2.06)	<0.001
Diabetes	1.48 (1.07, 2.05)	0.019	1.65 (1.10, 2.48)	0.015
Hypertension	1.18 (0.88, 1.57)	0.266		
Coronary heart disease	1.21 (0.76, 1.91)	0.424		
Heart failure	1.34 (0.82, 2.20)	0.239		
Cerebrovascular disease	1.46 (0.93, 2.29)	0.098	1.09 (0.61, 1.92)	0.778
Thrombosis	1.38 (0.92, 2.09)	0.121		
Malignancy	1.61 (0.89, 2.92)	0.113		

Data are presented as the odds ratio with the conﬁdence interval in parenthesis. OR, odds ratio; CI, conﬁdence interval.

**Figure 1:**
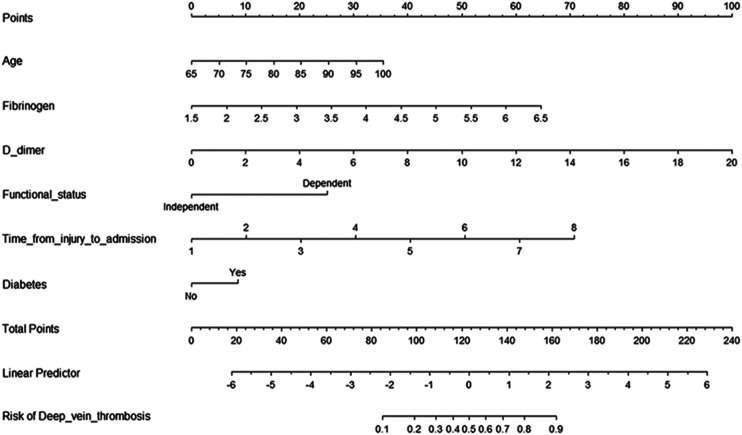
**Predictive nomogram for deep venous thrombosis**. To use the nomogram, the points corresponding to each prediction variable were obtained, then the sum of the points was calculated as the total score, and the predicted risk corresponding to the total score was the probability of DVT.

**Figure 2:**
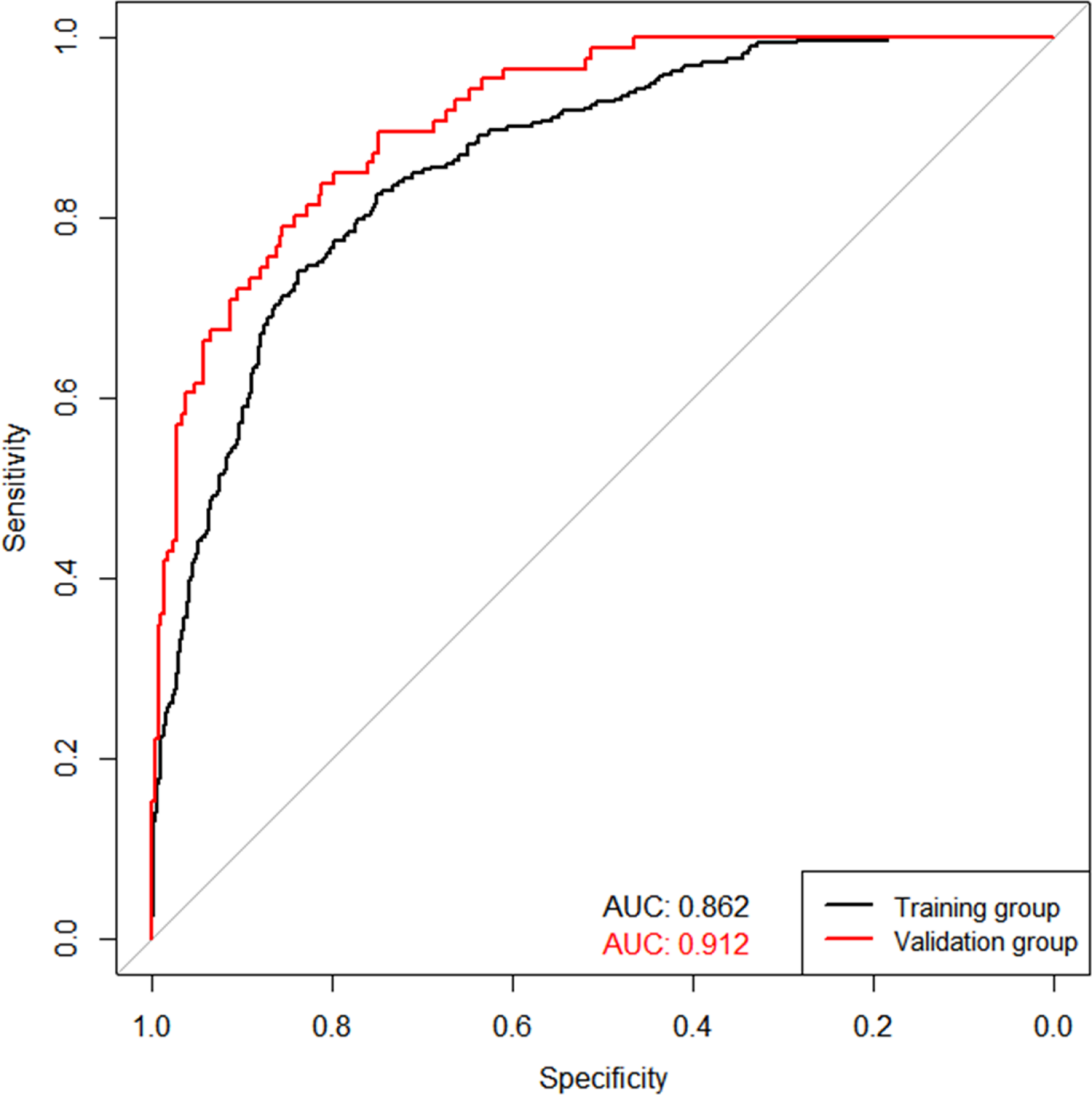
Example prediction nomogram for risk of deep venous thrombosis in a patient.

Then, we built a nomogram to predict DVT, including six independent risk factors based on multivariate logistic regression analysis (Figure 1). Predictive model: logit(DVT) = −13.84295 + 0.05758*age + 0.73397*fibrinogen + 0.28388*D-dimer + 0.57399*time from injury to admission + 1.42182*functional status + 0.48289*diabetes. According to the nomogram, the corresponding points of each predictor variable were obtained, the sum of the points was calculated as the total score, and the predicted risk corresponding to the total score was the probability of DVT. For example, an 85 years old patient (20 points) with fibrinogen at 4.5 g/L (39 points), d-dimer at 6 *μ*g/ml (30 points), the time from injury to admission was 2days (10 points), the functional status was dependent (25 points) and had a history of diabetes (8 points). The cumulative score of the various prediction indicators was 20 + 39 + 30 + 10 + 25 + 8 = 132, and the corresponding predicted risk of DVT was 0.62 (62%) (Figure 2).

**Figure 3:**
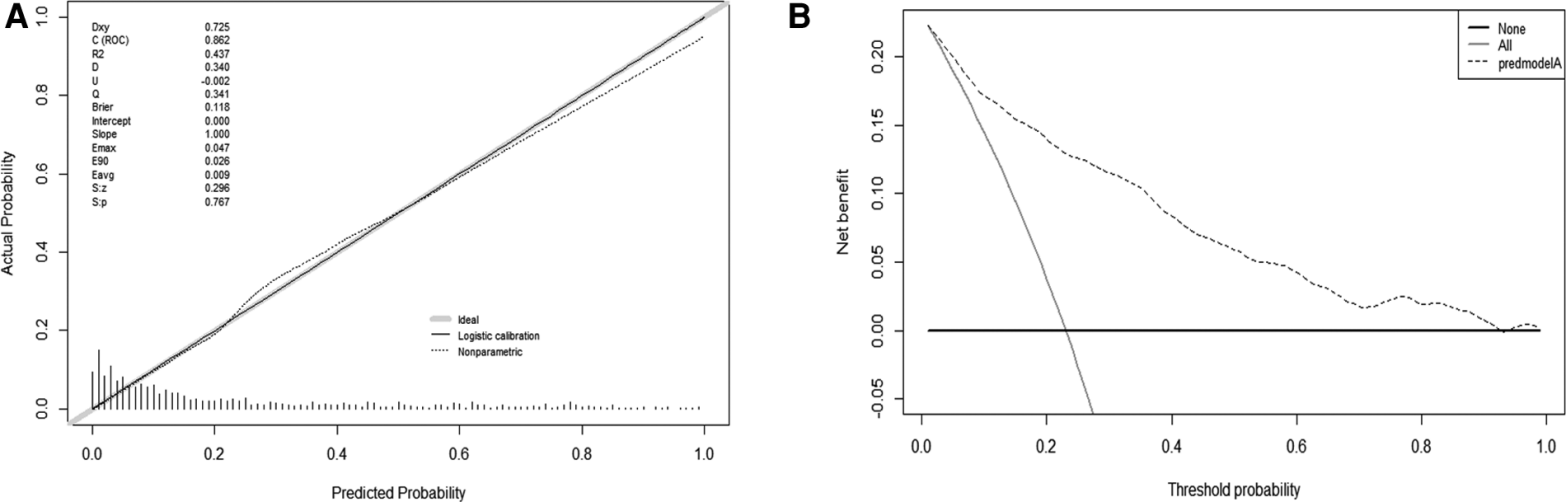
ROC curves for validating the discrimination power of the nomogram prediction model. (training group AUC = 0.862, validation group AUC = 0912).

**Figure 4:**
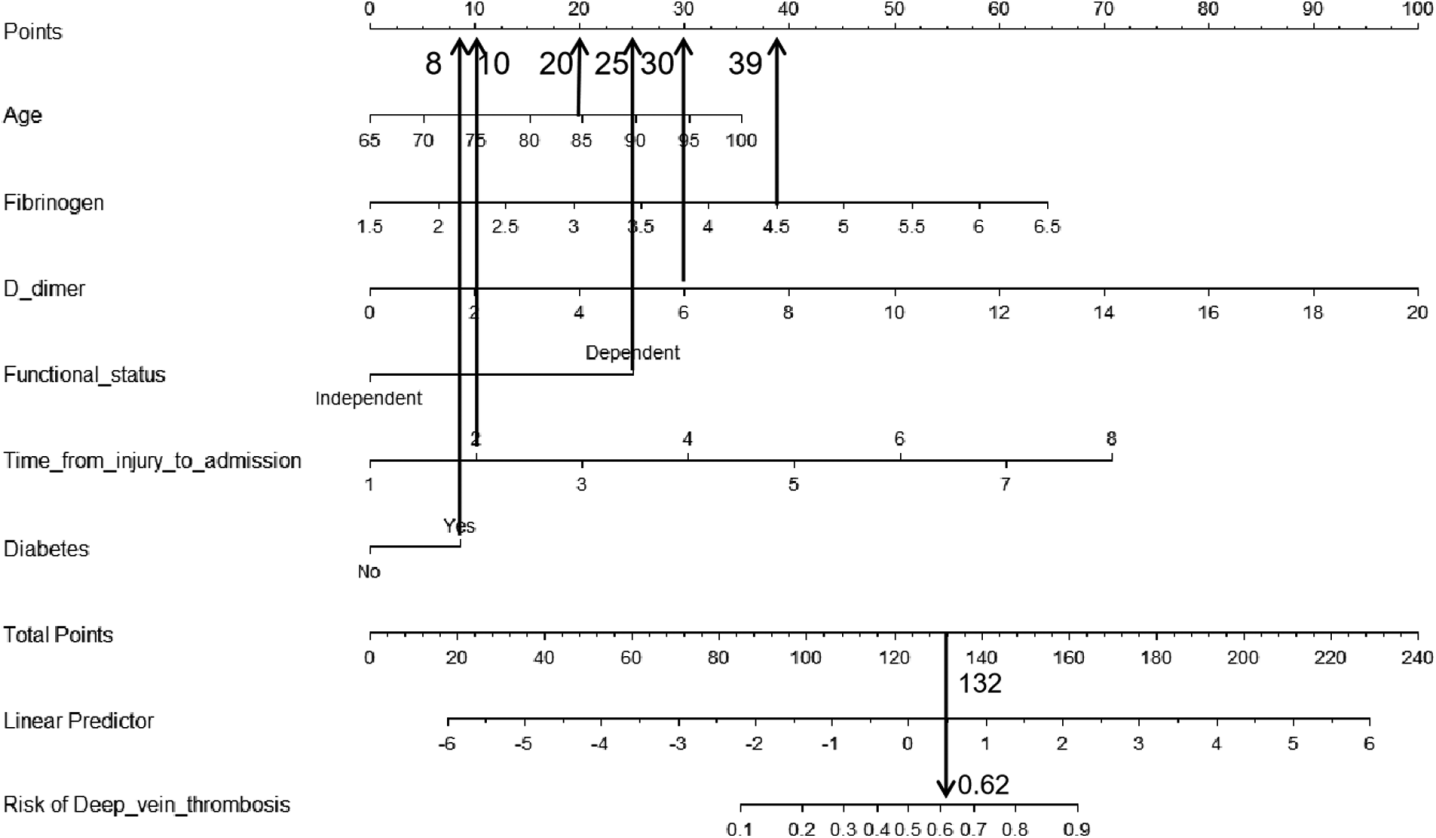
Calibration and decision plot of the nomogram for the probability of deep venous thrombosis. (A) Calibration plot. (B) Decision plot.

The validation of the model was based on discrimination and calibration. We plotted the ROC curve of the predictive model and calculated the AUC value. The AUC values for DVT of the training and validation group were 0.862 and 0.912, proving that this nomogram model has good discriminative power (Figure 3). The *P* value of Hosmer-Lemeshow test was 0.767, also indicating that this nomogram model had excellent calibration ability (Figure 4A). We hypothesize that the patient's treatment decision will be determined if the predicted probability exceeds the defined threshold, while the predicted probability below the threshold will lead to other decisions. Therefore, we evaluated the net benefit of this nomogram model to decision making through the decision analysis curve. The result showed that the nomogram was applicable when the threshold was in the range of 0 to 0.93 (Figure 4B).

## Discussion

Femoral intertrochanteric fracture is one of the most common fractures in the elderly, and the incidence of DVT after injury has been reported to range from 11.1 to 58.72% (18–21). There are too many reasons for thrombosis after trauma, including hypercoagulable state after fracture, hemostasis treatment, surgery and comorbidities (22–25). In our study, we found that the incidence of DVT during hospitalization was 23.02%. Age, fibrinogen, D-dimer, time from injury to admission, functional status and diabetes were considered as independent risk factors for DVT.

Age is a well-known risk factor for thrombosis in fracture patients, which has been proven by extensive studies. Wang et al. found that among patients with pelvic and acetabular fractures, the incidence of DVT was 71.4% in patients over 60 years old, which was much higher than that in patients under 60 years old (22.9%; *P* = 0.014) (26). A prospective cohort trial of the distal femur closed fracture conducted by Zhang et al. showed that age of ≥ 65 years of age (OR = 4.390; *P* = 0.002) was an independent risk factor of preoperative DVT (27). In our study, the age of patients in the DVT group (84.11 ± 5.91 years) was statistically higher as compared to that of the non-DVTs group (78.82 ± 7.25 years, OR = 1.07).

Fibrinogen is a protein synthesized in the liver and involved in blood clotting, accelerating the formation of blood clots. D-dimer is a fibrin degradation product, which increases as the fibrinolytic system becomes hyperactive. A retrospective study showed that fibrinogen and D-dimer levels was a good predictor of postoperative thrombosis after lower limb fractures (28). In addition, fibrinogen was considered as an independent risk factor for DVT after ankle fracture (29). Isolated calcaneal fracture patients had a low incidence of DVT, but elderly patients with delayed admission and elevated plasma D-dimer levels had a significantly increased risk (30). Similarly, our results showed that the OR of fibrinogen and D-dimer were 2.09 and 1.33.

Many articles had shown that among hip fracture patients undergoing surgery, increased waiting time was associated with an increased risk of mortality and other complications (31,32). Advocating early surgery could help patients recover their motor functions as soon as possible and reduced bed-ridden complications. We found that the time from injury to admission of patients in the DVT group (2.28 ± 1.34 days) was statistically longer as compared to that of the non-DVTs group (1.55 ± 0.91 days, OR = 1.78). A Korean study showed that delayed hip fracture surgery resulted in a higher incidence of VTE in patients undergoing routine prevention, however, intensive prevention programs successfully prevented postoperative symptomatic VTE, including pulmonary embolism (33). In addition, we found that patients with functionally dependent status were more likely to develop DVT. Such patients were often older, atherosclerotic, and long-term bedridden, which might be the main reason.

Most epidemiological studies have shown that patients with diabetes have an increased risk of DVT. Because there are high levels of circulating particles in diabetic patients, which are markers of systemic inflammation and procoagulant state, increasing the risk of thrombosis. The incidence of VTE in patients with diabetes after total hip (THA) or total knee anthroplasty (TKA) was significantly higher than that of patients without diabetes (34,35).

However, there were some limitations in our work. First of all, this study was a retrospective study. Although it was a dual center, the sample size of selected cases was small. Second, the most obvious was the reliance on the quality of medical record data. Third, we only investigated the incidence of DVT in the hospital. Since some DVTs occured after discharge from the hospital, our study might underestimate the true incidence of postoperative DVT. Future prospective randomized controlled trials should be further studied.

## Conclusion

In conclusion, we found that age, fibrinogen, D-dimer, time from injury to admission, functional status and diabetes were the independent risk factors for DVT. This nomogram model can be used to predict the probability of preoperative DVT in elderly patients with femoral intertrochanteric fracture and guide physician in perioperative thrombosis management.

## Data Availability

The raw data supporting the conclusions of this article will be made available by the authors, without undue reservation.
